# Dahl (S × R) Rat Congenic Strain Analysis Confirms and Defines a Chromosome 17 Spatial Navigation Quantitative Trait Locus to <10 Mbp

**DOI:** 10.1371/journal.pone.0058280

**Published:** 2013-02-28

**Authors:** Victoria L. Herrera, Khristine A. Pasion, Glaiza A. Tan, Nelson Ruiz-Opazo

**Affiliations:** Section of Cardiovascular Medicine, Department of Medicine, Boston University School of Medicine, Boston, Massachusetts, United States of America; Université Pierre et Marie Curie, France

## Abstract

A quantitative trait locus (QTL) linked with ability to find a platform in the Morris Water Maze (MWM) was located on chromosome 17 (*Nav-5* QTL) using intercross between Dahl S and Dahl R rats. We developed two congenic strains, S.R17A and S.R17B introgressing Dahl R-chromosome 17 segments into Dahl S chromosome 17 region spanning putative *Nav-5* QTL. Performance analysis of S.R17A, S.R17B and Dahl S rats in the Morris water maze (MWM) task showed a significantly decreased spatial navigation performance in S.R17B congenic rats when compared with Dahl S controls (P = 0.02). The S.R17A congenic segment did not affect MWM performance delimiting *Nav-5* to the chromosome 17 65.02–74.66 Mbp region. Additional fine mapping is necessary to identify the specific gene variant accounting for *Nav-5* effect on spatial learning and memory in Dahl rats.

## Introduction

One cognitive task frequently utilized to evaluate learning and memory performance in rodents is the hippocampus-dependent Morris water maze (MWM) test of spatial navigation [1], [2]. In this test the subject is placed in a large tank of water and is required to learn to swim to a small escape platform that is submerged below the surface of the water in a fixed spatial location. Placing the subject at different start locations around the tank ensures that it must navigate to the platform by the flexible use of distal visual cues placed about the room (e.g. cabinets, posters etc.).

Spatial navigation performance exhibits age-dependent decline in many species, including in humans [3]–[5], monkeys [6], [7], rats [8], [9] and mice [10] and hippocampal dysfunction has been reported as one of the earlier hallmarks of Alzheimer’s disease [11]. Thus, standard intercross linkage analysis aiming to identify typical genetic variations underlying strain differences in spatial navigation could help to establish a paradigm for investigation in humans.

We have recently reported the first genome-wide scan for quantitative trait loci (QTLs) affecting spatial learning and memory in Dahl rats [12]. We detected nine spatial navigation (*Nav*) QTLs on chromosomes 1, 2, 3, 8, 9, 11, 17, 20 and X affecting spatial learning and memory with various levels of significance [12]. The *Nav-5* QTL region on chromosome 17 (58–80 Mbp) was detected with the highest significant linkage (LOD 5.3) explaining 13% of total trait variance [12]. Thus, the present study was undertaking to 1) confirm the presence of one *Nav* QTL in this chromosome 17 region, and 2) delimit more precisely the chromosomal region harboring this *Nav-5* QTL.

## Results

**Figure 1:**
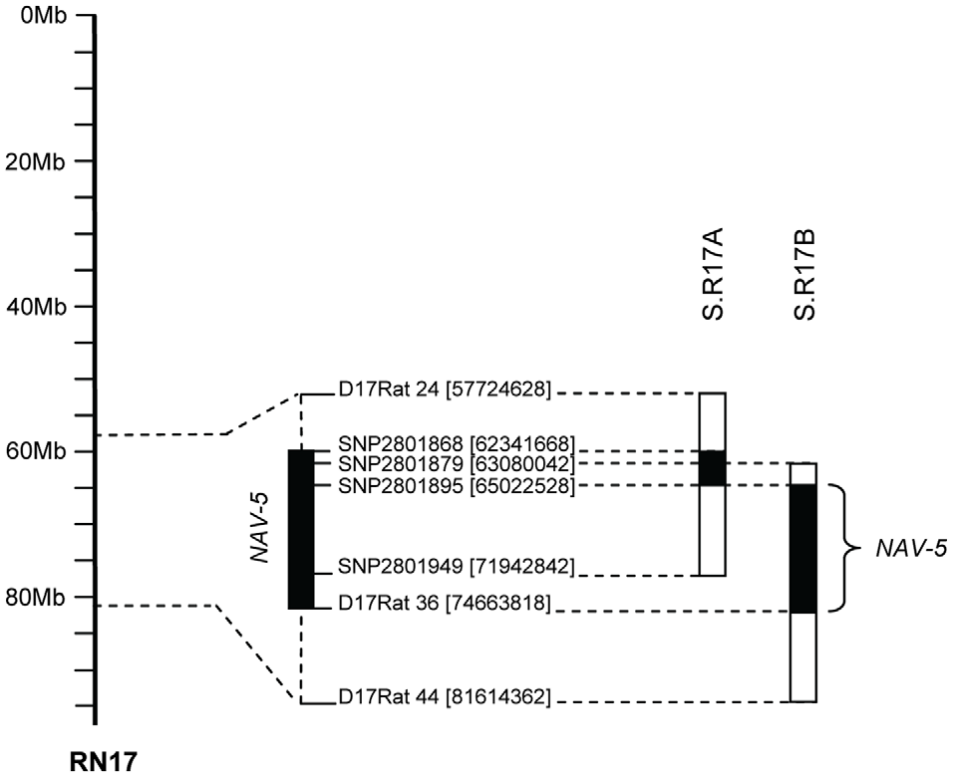
Congenic analysis of *Nav-5* QTL region on chromosome 17. On the left of the figure is shown the relevant region of the physical map of rat chromosome 17. Values in parenthesis next to the marker names denote physical locations in base pairs. The mapped *Nav-5* QTL region of approximately 9.64 Mbp is noted (to the right). Congenic strains are shown as solid bars (representing the Dahl R introgressed fragments) flanked by open bars (representing the putative regions of recombination).

To substantiate the existence of *Nav-5* QTL in the chromosome 17 58–80 Mbp region, we transferred two Dahl R chromosomal segments spanning the *Nav-5* QTL onto the Dahl S genetic background (shown in Figure 1). We implemented a “speed congenic” strategy towards the development of highly inbred S.R17A and S.R17B (Figure 1) congenic lines. At back-cross six we established homozygous congenic lines for assessment of navigational performance on the Morris water maze (MWM) task. At BC6 S.R17A was >99.85% of Dahl S genetic background and S.R17B >99.70% of Dahl S genetic background.

**Figure 2:**
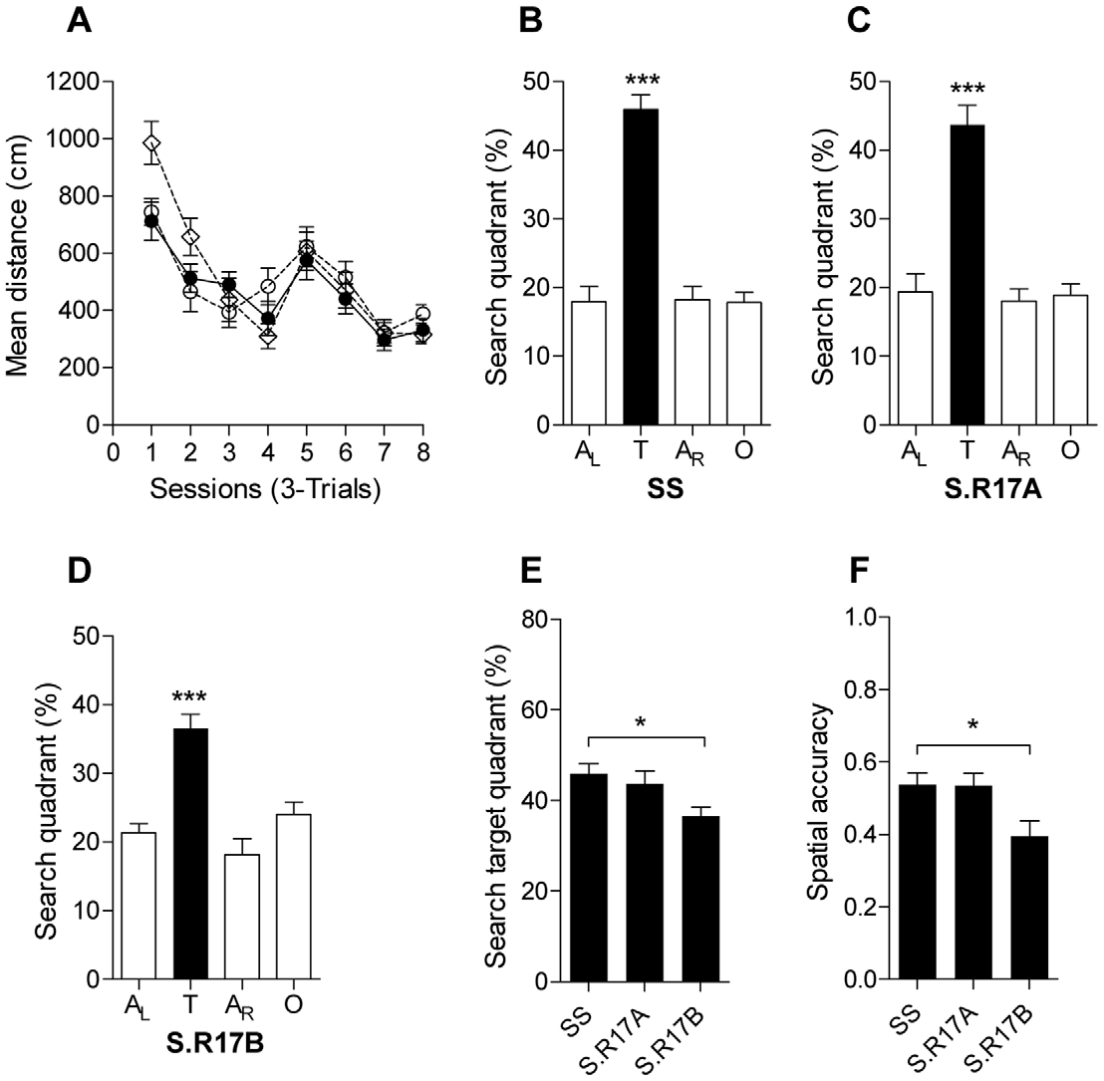
Testing of spatial learning and memory in Dhal S, S.R17A and S.R17B congenic male rats (A) Acquisition performance measured as mean distance in cm during the 24 trials (•, Dahl S; ◊, S.R17A; ○, S.R17B). Percentage distance traveled in quadrants (B, C, D, E) and spatial accuracy performance (F) during the probe trial after completion of Morris water maze training. Quadrants are: target (T), opposite (O), adjacent right (A_R_) and adjacent left (A_L_). *P = 0.02, ***P<0.001. Data represent means ± s.e.m. (one-way ANOVA followed by Holm-Sidak’s test for quadrant occupancy in the probe trial of the Morris water maze task and for spatial accuracy performance).

Measurements of spatial learning in the MWM task revealed equivalent acquisition performance among all three groups (Figure 2A, *F*_2,360_ = 1.39, *P*>0.24). In the probe trial for spatial memory all three groups exhibited target selectivity showing enhanced preference for the target quadrant over other quadrants (Figure 2B, Dahl S rats: *P*<0.001; Figure 2C, S.R17A rats: *P*<0.001; Figure 2D, S.R17B rats: *P*<0.001). However, direct comparison of selectivity for the target quadrant in the probe trial shows better performance of Dahl S controls compared with S.R17B subjects (Figure 2E, *P* = 0.02) and equivalent performance when compared with S.R17A subjects (Figure 2E, *P* = 0.79). Consistently, Dahl S rats showed increased spatial accuracy performance when compared with S.R17B subjects (Figure 2F, *P* = 0.02), corroborating their superior search accuracy for the hidden platform. Navigational performance in S.R17A congenic rats did not differ from Dahl S controls (Figure 2F, P = 0.96) demonstrating absence of genes affecting spatial learning and memory in this chromosomal segment, thus delimiting the chromosomal region to <10 Mbp (65.02–74.66 Mbp) that contains the gene underlying *Nav-5*.

## Discussion

Our results obtained in the present study confirmed the existence of *Nav-5* localizing to a single chromosome 17 congenic segment. Our congenic analysis maps *Nav-5* QTL between 65.02–74.66 Mbp (Figure 1) on chromosome 17. Importantly, the observed effect on navigational performance upon introgression of a Dahl R congenic fragment is consistent with expected directionality of spatial navigation effects; worsening performance in spatial learning and memory in S.R17B congenic rats [12].

Inspection of the chromosome 17 65–75 Mbp region encompassing *Nav-5* QTL shows 77 annotated genes. One annotated gene, LOC689258 [similar to Serine/threonine-protein kinase MARK1 (MAP/microtubule affinity-regulating kinase 1)] at 67.94 Mbp, could be a candidate for the observed effects of *Nav-5* QTL on spatial navigation. MARK1 has been shown to regulate microtubule assembly, neuronal differentiation and tau toxicity [13] an event implicated in late-onset Alzheimer’s disease. More recently, the MARK1-tau axis has been shown to play a critical role in mediating the toxic effects of Aβ on synapses and dendritic spines [14], neurodegenerative processes broadly associated with Alzheimer’s disease.

Our study demonstrates the existence of a QTL on chromosome 17 65–75 Mbp region that affects spatial learning and memory in Dahl S rats. The successful genetic isolation of the chromosome 17 *Nav-5* QTL present in S.R17B congenic line will form the basis to further fine map this QTL region to <0.1 Mbp via sub-strain construction and eventually identify the specific gene variant underlying this QTL. Identification of genes that influence spatial navigation in rats could help to establish a paradigm for investigation of similar pathways pertinent to age-dependent cognitive decline and Alzheimer’s disease in humans.

## Materials and Methods

### Ethics Statement

This study was performed in strict accordance with the recommendations in the Guide for the Care and Use of Laboratory Animals of the National Institutes of Health. The protocol was approved by the Committee on the Ethics of Animal Experiments of Boston University School of Medicine (Permit Number: AN-13926).

### Strains

All rats utilized in this study were bred in-house. Inbred Dahl S/jrHsd and Dahl R/jrHsd rats were obtained from Harlan (Indianapolis, Indiana). We transferred two Dahl R chromosomal segments spanning *Nav-5* onto the Dahl S genetic background. We implemented a “speed congenic” strategy [15], [16] to develop the two congenic lines. For this purpose we first produced a (Dahl S × Dahl R) F1 progeny followed by generation of an F1 × Dahl S backcross (BC1) population. We selected *Nav-5* “carriers” from 300 BC1 subjects by genotyping the BC1 male progeny with flanking markers of the chromosomal segments planned to be transferred. For S.R17A; sr heterozygous at SNP2801868 and SNP2801895, and ss homozygous at nearby flanking markers, i.e. D17Rat24 and SNP2801949. For S.R17B; sr heterozygous at SNP2801895 and D17Rat36, and ss homozygous at nearby flanking markers, i.e. SNP2801879 and D17Rat44. We then produced 20 BC2 male subjects per congenic line and proceeded to screen subjects with 85 informative SNPs. One “best” S.R17A male breeder containing 97.6% Dahl S genetic background and one “best” S.R17B male breeder containing 95.2% Dahl S genetic background were chosen to continue with the inbreeding program. Back-crosses were performed up to BC6 at which level we established homozygous congenic lines for blood pressure measurements and Morris Water Maze performance. SR17A was >99.85% of Dahl S genetic background and S.R17B >99.70% of Dahl S genetic background.

### Markers

We selected the following single nucleotide polymorphisms (SNPs) for congenic rat development from the rat genome data base (RGD): markers for S.R17A and S.R17B congenic fragments; D17Rat24, SNP2801868, SNP2801879, SNP2801895, SNP2801949, D17Rat36, D17Rat44. SNPs for implementation of “speed congenic” strategy, chr1: SNP2783361, SNP2783513, SNP2783573, SNP2783925, SNP2784073, SNP2784200, SNP2784723, SNP2784895, SNP2785046; chr2: SNP2785301, SNP2785499, SNP2785693, SNP2785860, SNP2786134, SNP2786276, SNP2786350, SNP2786619, SNP2786811, SNP2786979, SNP2787226; chr3: SNP2787599, SNP2787751, SNP2787947, SNP2788108, SNP2788217, SNP2788416; chr4: SNP2789191, SNP2789416, SNP2789717, SNP2789952, SNP2790223, chr5: SNP2790571, SNP2790733, SNP2790960, SNP2791234, SNP2791496, SNP2791711, SNP2791834; chr6: SNP2792065, SNP2792467, SNP2792754; chr7: SNP2793338, SNP2793565, SNP2793757, SNP2793904; chr8: SNP2794281, SNP2794450, SNP2794721, SNP2794865; chr9: SNP2795738, SNP2795947; chr10: SNP2796278, SNP2796474, SNP2796739, SNP2796966; chr11: SNP2797258, SNP2797443, SNP2797742; chr12: SNP2797924, SNP2798115; chr13: SNP2798475, SNP2798659, SNP2798785, SNP2798926; chr14: SNP2799254, SNP2799430, SNP2799825; chr15: SNP2800105, SNP2800195; chr16: SNP2800810, SNP2801108; chr17: SNP2801413, SNP2801584, SNP2801868, SNP2801948; chr18: SNP2802358, SNP2802507, SNP2802706; chr19: SNP2802997, SNP2803270; chr20: SNP2803540, SNP2803747; chrX: SNP2804065, SNP2804185, SNP2804233.

### Genotyping

DNA was extracted from tail biopsies using the QIAamp Tissue Kit (Qiagen, Valencia, CA). SNP genotyping was carried out on an Applied Biosystems 7900 Real-Time PCR System. All SNP assays (TaqMan assays) were procured from Applied Biosystems and were validated in our laboratory. All SSLPs were detected by PCR genotyping followed by denaturing polyacrylamide gel electrophoresis.

### Morris Water Maze Testing

The MWM task was performed as described [12], [17]–[19] using a 1.5 m diameter, circular water maze (filled with water at 25°C ±0.5), and a computer tracking system (Smart software program, Version 1.24, Panlab s.I., Barcelona, Spain). Distance was used to evaluate performance. Twelve swim trials were given per day (for 2 consecutive days). Animals were placed into the maze (at one of three randomized start positions located adjacent to the wall) and were allowed to traverse the maze in search of the escape platform. On each trial, a maximum swim time of 60 sec was imposed. Between trials, a 35 sec interval was imposed with the rat on the platform. At the end of the twenty-fourth trial, rats were removed to the home cage for 10 min, then the platform was removed (probe trial) and the rat was allowed to search for 1 minute. Sixteen Dahl S males, 16 S.R17A males and 16 S.R17B males were characterized in this MWM task for subsequent comparative analysis. Spatial accuracy was define as the ratio of % distance traveled in platform counter over the sum of % distance traveled over the four counters localized at analogous positions on corresponding quadrants (Spatial accuracy = % distance Tcounter/% distance Tcounter+% distance A_L_counter+% distance A_R_counter+% distance Ocounter).

### Statistical Analyses

We performed Two-Way ANOVA followed by Holm-Sidak test for acquisition performance comparisons and One-Way ANOVA followed by Holm-Sidak test for analysis of navigational performance on the probe trial.
